# Psychiatric In-Patients Are More Likely to Meet Recommended Levels of Health-Enhancing Physical Activity If They Engage in Exercise and Sport Therapy Programs

**DOI:** 10.3389/fpsyt.2018.00322

**Published:** 2018-07-20

**Authors:** Janine Ehrbar, Serge Brand, Flora Colledge, Lars Donath, Stephan T. Egger, Martin Hatzinger, Edith Holsboer-Trachsler, Christian Imboden, Nina Schweinfurth, Stefan Vetter, Markus Gerber

**Affiliations:** ^1^Department of Sport, Exercise and Health, University of Basel, Basel, Switzerland; ^2^Center for Affective, Stress and Sleep Disorders, Psychiatric Clinics of the University of Basel, Basel, Switzerland; ^3^Substance Abuse Prevention Research Center and Sleep Disorders Research Center, Kermanshah University of Medical Sciences, Kermanshah, Iran; ^4^Department of Intervention Research in Exercise Training, German Sport University Cologne, Cologne, Germany; ^5^Center for Integrative Psychiatry, Psychiatric Clinics of the University of Zürich, Rheinau, Switzerland; ^6^Psychiatric Services Solothurn, Solothurn, Switzerland; ^7^Private Clinic Wyss, Münchenbuchsee, Switzerland

**Keywords:** exercise, physical activity recommendations, psychiatric patients, sport, in-patients, psychiatry, Switzerland

## Abstract

**Background:** People with mental disorders engage in sedentary behaviors more often than their healthy counterparts. In Switzerland, nearly all psychiatric hospitals offer structured exercise and sport therapy as part of their standard therapeutic treatment. However, little is known about the degree to which psychiatric patients make use of these treatment offers. The aim of this study is to examine, in a sample of psychiatric in-patients (a) how many participate in the structured exercise and sport therapy programs offered by the clinic, (b) how many engage in exercise and sport activities on an individual basis, and (c) how many meet recommended levels of health-enhancing physical activity during their stay at the clinic. Furthermore, we examine whether those who engage in exercise and sport activities are more likely to meet internationally accepted physical activity recommendations.

**Methods:** 107 psychiatric in-patients (49% women, M_age_ = 39.9 years) were recruited at three psychiatric clinics in the German-speaking part of Switzerland. All participants were engaged in treatment and received usual care. Based on accelerometer data, participants were classified as either meeting or not meeting physical activity recommendations (≥150 min of moderate-to-vigorous physical activity per week). Participation in structured and individually performed exercise and sport activities was assessed with the Simple Physical Activity Questionnaire.

**Results:** In total, 57% of all patients met physical activity recommendations. 55% participated in structured exercise and sport therapy activities, whereas only 22% of all patients engaged in exercise and sport activities independently. Psychiatric patients were significantly more likely to meet recommended levels of health-enhancing physical activity if they engaged in at least 60 min per week of structured exercise and sport therapy or in at least 30 min of individually performed exercise and sport activity.

**Conclusions:** Given that prolonged immobilization and sedentary behavior have harmful effects on patients' physical and mental well-being, promoting exercise and sport activities is an important endeavor in psychiatric care. Clinics currently succeed in involving between 50 and 60% of all patients in sufficient physical activity. While this is encouraging, more systematic efforts are needed to ensure that all patients get enough physical activity.

## Introduction

In Europe, according to Wittchen and Jacobi (1), almost every second person suffers from a psychiatric disorder once in their lifetime. More recent data has shown that more than one third of the extended EU population develops at least one mental disorder per year (2). According to the World Health Organization (3, 4), the global burden associated with mental disorders is considerable, both for the sufferers, their family members, and society in general. In Switzerland, mental disorders are responsible for 14% of all disability-adjusted life years (1 DALY = one lost year of “healthy” life), coming third after cancer and musculoskeletal disorders, and even before cardiovascular diseases (5). Moreover, mental disorders are among the leading causes of disability in Switzerland, with 46% of all benefit claims being attributable to mental disorders (5). Finally, Maercker et al. (6) showed that in 2010 the total costs incurred by mental disorders amounted to 14.5 billion Euro (≈ 1900 EURO per inhabitant per year), with Switzerland ranked markedly above the European average.

Based on findings from several large-scale prospective studies, there is evidence that physical activity has the potential to prevent mental health issues (7). For instance, Brown et al. (8) showed that higher levels of previous, current, and habitual physical activity were associated with a lower risk of developing depressive symptoms at the 5-year follow-up. Mikkelsen et al. (9) found that women with low initial levels of physical activity were significantly more likely to develop depression during the 26-year follow-up period. Moreover, Lindwall et al. (10) showed in a 6-year longitudinal study that changes in physical activity are related to changes in depression, anxiety, and burnout symptoms. That is, if participants' physical activity levels increased over time, the frequency of self-reported symptoms decreased, and vice versa.

Previous research has also shown that people with mental disorders more often engage in sedentary behaviors than those who have no mental disorder, and are therefore less likely to meet internationally accepted standards for health-enhancing (that is, moderate-to-vigorous) physical activity (11, 12). For instance, Schuch et al. (13) showed that among patients with major depressive disorder, more than two thirds (68%) were not sufficiently physically active (<150 min of moderate-to-vigorous physical activity per week). Moreover, patients had a significantly increased likelihood of not meeting recommended levels of physical activity compared to healthy controls (OR = −1.50, 95% CI: −1.10 to −2.10). Lower physical activity levels compared to healthy controls were also found in patients with schizophrenia disorders (14).

In the wake of this research, physical activity and exercise have gained increasing recognition among health professionals, and are now recommended by several health foundations as a first- or second-line strategy in the treatment of psychiatric disorders (15–17). In recent years, a number of studies have shown that exercise therapy can make an important contribution to the improvement of psychiatric symptoms (18–20), either as an adjunct (21, 22), or stand-alone therapy (23). Research has also shown that exercise therapy can be equally as effective as pharmacological treatment (24), and is even effective in patients with treatment-resistant disorders (25).

While prior investigations have suggested that one single exercise episode can have positive effects on patients' well-being (26, 27), studies also show that repeated participation in exercise therapy has a clinically relevant impact on psychiatric patients' cardiorespiratory fitness levels (28). The latter is important because cardiorespiratory fitness is associated with lower risk for cardiovascular diseases and cardiovascular disease-related mortality (29, 30), conditions to which patients with psychiatric disorders are particularly susceptible (31, 32). Moreover, regular physical activity and exercise therapy have the potential to attenuate some of the negative side effects associated with pharmacological treatment such as excessive weight gain (33). Furthermore, previous research has shown that prolonged immobilization (e.g., through extended bed rest) can have harmful effects leading to a rapid reduction in muscle mass, bone mineral density, and impairments in other body systems (34–36).

Given the aforementioned, it seems likely that it could be beneficial to get psychiatric patients involved in exercise and sport activities on a regular basis (37). Although translation of empirical evidence into routine clinical practice can be a slow process (38), Brand et al. (39) showed that in the German-speaking part of Switzerland, nearly all psychiatric hospitals already now offer structured exercise and sport therapy (97%) or physiotherapy (85%) as part of their standard therapeutic treatment. However, clinical managers roughly estimated that only about a quarter of all psychiatric patients make use of these treatment offers, although this figure has not been empirically investigated. The extent to which patients exercise on an individual basis while in treatment was also not assessed. Therefore, the main purpose of the present study was to collect empirical data on psychiatric in-patients in order to examine how many of them (a) participate in structured exercise and sport therapy programs offered by the clinic and lead by a member of staff, (b) engage in exercise and sport activities on an individual basis, and (c) meet recommended levels of health-enhancing physical activity during their stay at the clinic. Furthermore, we wanted to find out whether those who engage in exercise and sport activities are more likely to meet internationally accepted recommendations for health-enhancing physical activity.

## Methods

### Participants and procedures

Potential participants were recruited at three psychiatric clinics in the German-speaking part of Switzerland. All participants were engaged in treatment at the relevant sites at the time of recruitment to the study. All participants were receiving usual care involving any combination of pharmacological, psychotherapeutic, and group-based treatments. They were identified and referred to the research team by the treating clinicians at each of the three sites. Participants were approached by a researcher not involved in direct care, and were informed that the decision to participate would not have any impact on the treatment they received. The researcher then explained details of the study methodology and asked all those interested to provide written informed consent. Prior to the start of the study, ethical approval was sought from the local ethics committee (Ethical commission of North-western and Central Switzerland, EKNZ Nr. 2016-01547) to ensure that all procedures were in line with current Swiss legal requirements. Moreover, all procedures met the ethical requirements defined in the declaration of Helsinki and its later amendments.

Inclusion criteria were: (a) aged 18–65 years, (b) current in-patient of a psychiatric clinic, and (c) meeting ICD-10 criteria for a mental disorder. Exclusion criteria were: (a) evidence of significant cardiovascular, neuromuscular, or endocrine disorders limiting regular ambulation (as per the American College of Sports Medicine absolute contraindications to exercise) (40), (b) current diagnosis of anorexia nervosa or bulimia, (c) current diagnosis of an organic brain disorder, and (d) estimated MoCA (Montreal Cognitive Assessment; www.mocatest.org) score < 26.

**Figure 1:**
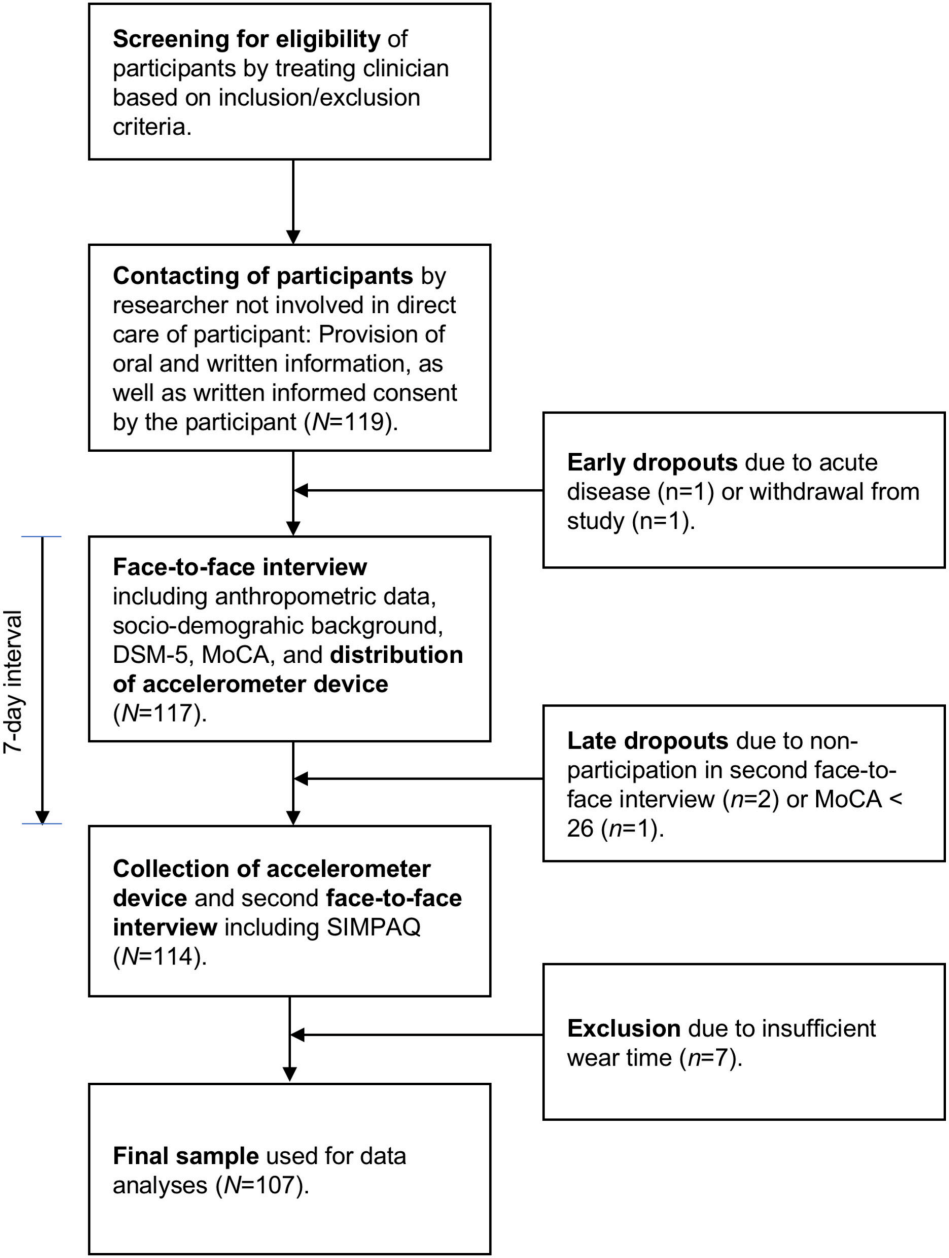
Graphical overview of study design, data assessment and participant flow chart.

Based on a power size calculation, between 52 and 128 patients were needed to detect either strong- or moderate-sized differences in physical activity levels between patients who participate vs. do not participate in structured exercise and sport therapy programs (based on G^*^Power 3.1: ANOVA: fixed effects, omnibus, effect size: f = 0.25 (moderate effect) or 0.40 (strong effect), α = 0.05, Power = 0.80). As shown in Figure 1, 119 participants were willing to participate in the study. Among these, 117 participated in the initial data assessment. Two participants dropped out prior to the second assessment session, and one participant had to be excluded due to severe cognitive impairments (MoCA score < 26). Seven participants had to be excluded because they did not meet minimal standards for accelerometer wear-time (see below for more details), leaving a final sample of 107 patients (52 women, 55 men, M = 39.9 years, *SD* = 12.2).

From the 107 participants, 23% (*n* = 25) were diagnosed with schizophrenia spectrum disorder, 18% (*n* = 19) with bipolar disorder, 66% (*n* = 71) with major depressive disorder, 13% (*n* = 14) with anxiety disorder, 1% (*n* = 1) with obsessive compulsive disorder, 12% (*n* = 13) with trauma or stress-related disorder, 11% (*n* = 12) with a substance abuse disorder, and 10% (*n* = 11) with another disorder (e.g., adjustment disorder, dissociative disorder, attention deficit hyperactivity disorder). While 52% (*n* = 56) were diagnosed with one disorder, 40% (*n* = 42) had a double diagnosis, and 8% (*n* = 9) of the patients were diagnosed with three disorders. Almost 70% (*n* = 73) used antidepressants, 55% (*n* = 59) antipsychotics, 33% (*n* = 35) mood stabilizers, 30% (*n* = 32) sedative substances, and 21% (*n* = 22) other drugs (e.g., antidiabetics, diuretics); all at therapeutic dosages. In total, 63% (*n* = 67) identified themselves as smokers. Furthermore, 39% (*n* = 42) were living in a relationship, whereas 61% (*n* = 65) were living alone (single, divorced, separated, widowed). On average, participants reported 12.7 years of education (*SD* = 2.7), and 51% (*n* = 54) reported that they were working before they entered the clinic (on average, 30.6 h, *SD* = 12.5, per week before admission to the clinics).

### Data assessment

Data assessment took place between September 2016 and April 2017. The research officer directly obtained demographic and diagnostic information via a demographic and diagnostic data sheet that includes sex, living situation, education, employment status, smoking status, psychiatric diagnoses, somatic co-morbidities, and medication usage. Moreover, the research officer directly measured anthropometric outcomes (height and weight). Further assessments included: (a) the DSM-5 Self-Rated Level 1 Cross-Cutting Symptom Measure—Adult (DSM-5); and (b) the MoCA Test. At the end of the assessment session, a tri-axial accelerometer device (ActiGraph GT3X+) was given to all participants to objectively assess physical activity during the following 7 days. The researchers collected the accelerometer at the end of this time, and further assessed information about the participants' physical activity during the past 7 days via the Simple Physical Activity Questionnaire (SIMPAQ). More information about all measures is provided below.

### Cross-cutting psychiatric symptoms

We used the DSM-5 Self-Rated Level 1 Cross-Cutting Symptoms Measure for adults to assess the presence and severity of 13 psychiatric symptom domains that cut across diagnostic boundaries (41). This instrument is not a diagnostic or screening tool, but has been developed to give clinicians quantitative, simple, and useful ratings of a patient's characteristics that may influence clinical decision-making. The Level 1 measure for adults consists of 25 items, which are answered on a 5-point Likert scale from 0 (none/never) to 4 (severe/almost daily), referring to the past 2 weeks. Higher scores reflect higher frequency of occurrence or greater degree of severity. The DSM-5 approach proved to be feasible and clinically useful in multiple routine practice settings (42). The Cronbach's alpha in the present sample was 0.87.

### Depressive symptoms

We used the German version of the Beck Depression Inventory-II (BDI-II) to measure the severity of depressive symptoms (43). The BDI-II includes 21 items, covering a range of affective, behavioral, cognitive, and somatic symptoms that are indicative of unipolar depression (e.g., “I am so unhappy/sad that I can't stand it”). Four response options with increasing levels of depressive symptomatology are provided for each. Possible scores range from 0 to 63 with higher scores indicating more depressive symptoms. Adequate validity and reliability of the BDI-II have been shown previously (44). The Cronbach's alpha in the present sample was 0.92.

### Objectively assessed physical activity

We used the ActiGraph® wGT3X-BT accelerometer to objectively assess physical activity. Evidence of the validity and reliability of this light triaxial activity monitor to accurately capture physical activity has been documented previously (45). Participants wore the device continuously during the study period, except for activities taking place in water lasting longer than 30 min, or taking place below a one meter depth of water. The monitor was placed on a strap around the wrist of the non-dominant hand. The sample rate was 30 Hz, and raw data files were analyzed with the ActiLife Software (Version 6.13.2). Data were summed and stored in 15 s epoch lengths. Daily summed minute-by-minute data were categorized by cut-off values. The cut-off values developed by Kamada et al. (46) were used, which were < 2,000 vector magnitude counts per minute (VM cpm) for sedentary, 2000–7499 VM cpm for light physical activity, and ≥7500 VM cpm for moderate-to-vigorous physical activities. With triaxial accelerometers, vector magnitude is calculated from the three axes using the following formula: (x^2^ + y^2^ + z^2^)^1/2^. Non-wear time was determined using the Troiano (47) algorithm with default settings. Following Clemente et al. (48), days with ≥10 percent of non-wear time were considered non-valid and excluded. To be included, participants had to have at least five valid days, including ≥4 valid weekdays and ≥1 valid weekend day (only considering the 7 days prior to the telephone interview). Weekly scores were obtained by dividing the sum of all valid days through the number of valid days, and then multiplying by seven. The following indices were examined (in min per week): sedentary activity, light physical activity (LPA) and moderate-to-vigorous physical activity (MVPA). Moreover, the weekly number of steps was measured.

### Self-reported physical activity

Self-reported physical activity levels were assessed with the Simple Physical Activity Questionnaire (SIMPAQ). The SIMPAQ is a brief five-item tool, which comprehensively evaluates activity over the past 7 days including time in bed, sedentary time, time spent walking, time spent in exercise (by type), and time spent for other activities (49). The SIMPAQ refers to all domains of activity, including leisure time, domestic, work, and transport-related activities. Moreover, the SIMPAQ captures a 24-h period representative of the previous week. The following indices will be analyzed in the present study (in min/week): time in bed, sedentary time, time spent standing, time spent walking, other physical activities, and exercise.

### Statistical analyses

*M, SD*, skewness, and kurtosis were calculated as indicators for the descriptive statistics. Log binominal models were used to find out whether those patients who either take part in structured exercise and sport therapy or individually engage in exercise and sport activities are more likely to accomplish recommended levels of physical activity. Recommended levels of health-enhancing physical activity were defined according to the standards of the Centers for Disease Control and Prevention (www.cdc.gov), stating that for substantial health benefits, adults should do at least 150 min a week of moderate-intensity physical activity. Log binominal models are generalized linear models with a logarithmic link function and binomial distribution function. For common outcomes with prevalence rates of >10%, log binominal model is the recommended method for adjusted risk ratio (RR) estimation because the relative risk under the above mentioned circumstances can be overestimated using the odds ratios from logistic regression (50). Separate analyses were performed for (a) structured exercise and sport therapy activities, (b) individually performed exercise and sport activities, and (c) a combination of structured and individually performed exercise and sport activities. Moreover, two different cut-offs (30 vs. 60 min /week) were applied across all analyses. Finally, an uncontrolled model was compared with a model controlled for potential confounders (age, sex, education, smoking status, depressive symptoms, psychiatric symptom severity, walking, and other physical activities). Adjusted risk ratios (RR) in combination with the corresponding 95% confidence intervals (CI) are presented as estimates of effect measures. The level of significance was set at *p* < 0.05 across all analyses. All statistical tests were performed with SPSS® 24 (IBM Corporation, Armonk, NY, USA) for Apple Mac®.

## Results

### Descriptive statistics

Table 1 provides the descriptive statistics of the main study variables. Kolmogorov-Smironov and Shapiro-Wilk tests of normality showed that psychiatric symptom severity, depressive symptoms, and light physical activity were normally distributed (*p* > 0.05), whereas moderate-to-vigorous physical activity and all of the self-reported physical activity indicators were not. For actigraphy-based moderate-to-vigorous physical activity, walking, and other physical activities, the skewness (<2) and kurtosis (<7) were in the acceptable range (51), which was not the case for structured and individually performed exercise and sport activities. Therefore, these two variables were transformed into categorical variables, hereby distinguishing between participants with less (0) vs. equal or more (1) than 30 min of these activities per week. Additionally, an alternative categorization was made to distinguish between participants who engage in less vs. equal or more than 60 min per week of these activities.

**Table 1:** Descriptive statistics, psychometric properties of the main study variables.

	***M***	***SD***	**Range**		**Skew**	**Kurt**
			**Potential**	**Actual**		
**PSYCHIATRIC SYMPTOMS**						
Psychiatric symptom severity (DSM-5)	1.41	0.68	0–4	0–3.13	0.21	−0.55
Depressive symptoms (BDI-II)	20.19	11.39	0–63	0–52	0.40	−0.39
**ACTIGRAPHY**						
Light physical activity (min/week)	2253	616	0+	822–3228	0.31	0.15
Moderate-to-vigorous physical activity (min/week)	222	185	0+	7–1031	1.59	3.46
**SELF-REPORTED PHYSICAL ACTIVITY**						
Structured sport and exercise therapy (min/week)	72	111	0+	0–811	3.49	18.60
Individually performed sport and exercise (min/week)	28	67	0+	0–361	2.80	8.15
Walking (min/week)	731	324	0+	210–1260	0.48	−0.99
Other physical activities (min/week)	306	357	0+	0–1260	1.65	1.86

*DSM-5, DSM-5 Self-Rated Level 1 Cross-Cutting Symptom Measure for Adults; BDI-II, Beck Depression Inventory—II (revised version)*.

Based on a Mahalonobis D^2^ test, no multivariate outliers were detected. Moreover, no evidence of multicollinearity was found between the independent study variables (all VIP values < 3). Therefore, key assumptions were met in order to perform multiple regression analyses.

### Recommended physical activity levels

In the present sample, 57% (*n* = 61; 35 females, 26 males) of the patients met recommended physical activity levels, whereas 43% (*n* = 46, 17 females, 29 males) did not. A χ^2^-test showed that female patients (67%) were more likely to meet physical activity recommendations than male patients (47%), $\chi_{\left(1,107\right)}^{2}$ = 4.38, *p* < 0.05. Moreover, patients who met physical activity recommendations self-reported higher scores for (structured) exercise and sport therapy (*M* = 92 min/week, *SD* = 133), *F*_(1, 105)_ = 4.81, *p* < 0.05, η^2^ = .044, individually performed exercise and sport activities (*M* = 43 min/week, *SD* = 80), *F*_(1, 105)_ = 6.96, *p* < 0.01, η^2^ = 0.062, and walking (*M* = 796 min/week, *SD* = 308), *F*_(1, 105)_ = 5.82, *p* < 0.05, η^2^ = 0.052, than patients with insufficient physical activity levels (exercise and sport therapy: *M* = 46 min/week, *SD* = 63; individually performed sport and exercise activities: *M* = 9 min/week, *SD* = 38; walking: *M* = 647 min/week, *SD* = 327). No significant differences were found between patients who met vs. did not meet physical activity recommendations with regard to age, *F*_(1, 105)_ = 0.14, *p* = ns, education, *F*_(1, 105)_ = 0.03, *p* = ns, smoking status, $\chi_{\left(1,107\right)}^{2}$ = 1.66, *p* = ns, psychiatric symptom severity, *F*_(1, 105)_ = 0.02, *p* = ns, depressive symptoms, *F*_(1, 105)_ = 0.03, *p* = ns, or other physical activities, *F*_(1, 105)_ = 0.86, *p* = ns.

### Exercise and sport therapy

Table 2 shows that if patients engage in ≥ 60 min per week of exercise and sport therapy, they are more likely to meet recommended physical activity standards. Both in the uncontrolled model (RR = 2.62, 95% CI: 1.16; 5.93), and after controlling for potential confounders (RR = 2.45, 95% CI: 1.01; 5.94), the likelihood of being sufficiently physically active was more than two times higher. Table 2 also shows that a minimum of 60 min per week seemed necessary. While the likelihood of meeting physical activity recommendations was increased in patients with ≥30 min of sport and exercise therapy per week, they did not significantly differ from participants who engage in <30 min per week of exercise and sport therapy activities (95% CI in controlled model: 0.76; 4.31).

**Table 2:** Multivariate relationships between weekly time spent in structured sport and exercise therapy programs, before and after controlling for socio-demographic background and symptoms severity.

	**Minutes per week in exercise and sport therapyprograms**			
	≥**30 min**^a^		≥**60 min**^b^	
	**RR**	**95% CI**	**RR**	**95% CI**
**MODEL 1: UNCONTROLLED**				
Below	1		1	
Above	1.84	0.85; 3.98	**2.62**	**1.16; 5.93**
**MODEL 2: CONTROLLED FOR SOCIO-DEMOGRAPHIC BACKGROUND, SYMPTOM SEVERITY, WALKING, AND OTHER PHYSICAL ACTIVITIES**				
Below	1		1	
Above	1.80	0.76; 4.31	**2.45**	**1.01; 5.94**
**Potential confounders**				
Age	0.97	0.94; 1.01	0.97	0.94; 1.01
Sex	**2.46**	**1.02; 5.92**	**2.68**	**1.09; 6.57**
Education	0.94	0.78; 1.09	0.94	0.79; 1.11
Smoking status	0.70	0.28; 1.68	0.70	0.28; 1.72
BDI-II	0.99	0.93; 1.05	0.99	0.93; 1.05
DSM-5	1.42	0.54; 4.18	1.42	0.51; 3.99
Walking	1.00	0.99; 1.01	1.00	0.99; 1.01
Other activities	1.00	0.99; 1.01	1.00	0.99; 1.01

RR, Relative Risk Ratio; 95% CI, 95% confidence interval; BDI-II, Beck Depression Inventory – II (second version); DSM-5, DSM-5 Self-Rated Level 1 Cross-Cutting Symptom Measure for Adults. Significant associations are marked in bold.

a *n = 49 below (46%), n = 58 above (54%) ≥30 min/week cut-off*.

b *n = 63 below (59%), n = 44 above (41%) ≥60 min/week cut-off*.

### Individually performed exercise and sport activities

Table 3 shows that even 30 min per week of individually performed exercise and sport activities are associated with an increased likelihood of meeting recommended levels of physical activity. The RR was markedly increased both in the uncontrolled model (RR = 6.00; 95% CI: 1.65; 21.87), and after controlling for potential confounders (RR = 6.27; 95% CI: 1.58; 24.92). Table 3 further shows that the likelihood of being sufficiently physically active slightly increased if patients self-reported ≥60 min of weekly individually performed sport and exercise activity (controlled model: RR = 8.22; 95% CI: 1.61; 42.08).

**Table 3:** Multivariate relationships between weekly time spent in individually performed sport and exercise activities, before and after controlling for socio-demographic background and symptoms severity.

	**Minutes per week in individually performedsport and exercise activities**			
	≥**30 min**^a^		≥**60 min**^b^	
	**RR**	**95% CI**	**RR**	**95% CI**
**MODEL 1: UNCONTROLLED**				
Below	1		1	
Above	**6.00**	**1.65; 21.87**	**7.17**	**1.55; 33.20**
**MODEL 2: CONTROLLED FOR SOCIO-DEMOGRAPHIC BACKGROUND, SYMPTOM SEVERITY, WALKING, AND OTHER PHYSICAL ACTIVITIES**				
Below	1		1	
Above	**6.27**	**1.58; 24.92**	**8.22**	**1.61; 42.08**
**Potential confounders**				
Age	0.98	0.94; 1.02	0.98	0.94; 1.02
Sex	**2.50**	**1.02; 6.15**	**2.52**	**1.02; 6.23**
Education	0.93	0.79; 1.10	0.94	0.79; 1.11
Smoking status	0.68	0.27; 1.70	0.58	0.23; 1.46
BDI-II	0.99	0.94; 1.05	0.99	0.93; 1.06
DSM-5	1.40	0.49; 3.99	1.37	0.48; 3.94
Walking	1.00	0.99; 1.01	1.00	0.99; 1.01
Other activities	1.00	0.99; 1.01	1.00	0.99; 1.01

RR, Relative Risk Ratio; 95% CI, 95% confidence interval; BDI-II, Beck Depression Inventory – II (second version); DSM-5, DSM-5 Self-Rated Level 1 Cross-Cutting Symptom Measure for Adults. Significant associations are marked in bold.

a n = 86 below (80%), n = 21 above (20%) ≥30 min/week cut-off.

b *n = 86 below (84%), n = 17 above (16%) ≥60 min/week cut-off*.

### Combination of structured and individually performed exercise and sport activities

Table 4 shows that a total of 30 min of exercise and sport activities, either in the form of structured therapy or individually performed, was associated with an increased likelihood of meeting recommended physical activity standards (controlled model: RR = 2.37, 95% CI: 1.04; 5.78). The likelihood of meeting physical activity standards increased if ≥60 min per week was used as a cut-off (controlled model: RR = 3.05, 95% CI: 1.28; 7.27).

**Table 4:** Multivariate relationships between weekly time spent in either structured sport and exercise programs or individually performed sport and exercise activities, before and after controlling for socio-demographic background and symptoms severity.

	**Minutes per week in either exercise andsport therapy programs or individually performedsport and exercise activities**			
	≥**30 min**^a^		≥**60 min**^b^	
	**RR**	**95% CI**	**RR**	**95% CI**
**MODEL 1: UNCONTROLLED**				
Below	1		1	
Above	**2.39**	**1.08; 5.31**	**3.41**	**1.53; 7.64**
**MODEL 2: CONTROLLED FOR SOCIO-DEMOGRAPHIC BACKGROUND, SYMPTOM SEVERITY, WALKING, AND OTHER PHYSICAL ACTIVITIES**				
Below	1		1	
Above	**2.37**	**1.04; 5.78**	**3.05**	**1.28; 7.27**
**Potential confounders**				
Age	0.97	0.94; 1.01	0.98	0.94; 1.01
Sex	**2.40**	**1.05; 5.85**	**2.61**	**1.06; 6.43**
Education	0.91	0.77; 1.09	0.93	0.79; 1.10
Smoking status	0.68	0.28; 1.65	0.68	0.27; 1.70
BDI-II	0.99	0.93; 1.05	0.99	0.93; 1.05
DSM-5	1.44	0.51; 4.02	1.33	0.47; 3.76
Walking	1.00	0.99; 1.01	1.00	0.99; 1.01
Other activities	1.00	0.99; 1.01	1.00	0.99; 1.01

RR, Relative Risk Ratio; 95% CI, 95% confidence interval; BDI-II, Beck Depression Inventory – II (second version); DSM-5, DSM-5 Self-Rated Level 1 Cross-Cutting Symptom Measure for Adults. Significant associations are marked in bold.

a n = 41 below (38%), n = 66 above (62%) ≥30 min/week cut-off.

b *n = 54 below (50%), n = 53 above (50%) ≥60 min/week cut-off*.

## Discussion

The key finding of the present study is that psychiatric patients were more likely to meet recommended levels of health-enhancing physical activity (≥150 of moderate-to-vigorous physical activity per week) if they engaged in at least 60 min per week of structured exercise and sport therapy or in at least 30 min of individually performed exercise and sport activity. The relative risk ratios were of considerable magnitude, showing that participation in any exercise and sport activities greatly contributes to the achievement of recommended physical activity standards among psychiatric in-patients.

Our findings are in line with previous research showing that even small doses of exercise and sport can have positive effects among psychiatric patients. For instance, Hallgren et al. (52) found in a randomized controlled trial, in which patients with mild-to-moderate depression were randomized to supervised physical exercise or usual care by a physician (including cognitive behavioral therapy and antidepressant treatment), that the exercise group reported significantly lower depression severity at post-treatment. These differences were maintained until 12-month follow-up, although most patients only attended one exercise class per week. This finding corresponds well with a study in initially physically inactive patients suffering from job-related exhaustion disorder. In this study, Lindegård et al. (53) observed that patients who engaged in at least one exercise session per week after completion of 12 months of multimodal treatment showed a further decrease in psychiatric symptoms, whereas symptom severity started to rise again in patients who did not engage in any exercise activities. Thus, our findings expand these results by showing that even a small increase in self-reported physical activity plays a decisive role in whether or not psychiatric patients meet recommended levels of health-enhancing physical activity.

For several reasons, the finding that exercise and sport contribute to the accomplishment of recommended physical activity standards among psychiatric in-patients is important: First, our results suggest that both structured and individual exercise are effective in ensuring that patients meet these standards. This may be an initial indicator that the type of exercise itself is less important than engagement in exercise *per se*, albeit of sufficient intensity and duration. Our findings suggest that clinics which aim to promote the holistic health of their patients should offer both structured sessions and opportunities (such as equipment or local gym membership) to participants. Equally, it may be reassuring to patients to know that choosing a form of exercise that they prefer, rather than abstaining because they dislike the structured program being offered, can be equally positive for their health.

Second, previous research has shown that correlations between self-reported and objectively assessed exercise are only of moderate magnitude (54). In our study, this might mean that if a patient self-reports 60 min of structured exercise and sport therapy, only a limited amount of time might be spent in moderate-to-vigorous physical activity because time is lost for (low-intensity) warming-up, instruction, feedback, and cooling down. While self-reported intensity of individual activity is also open to criticism (see above), in our study, it appears that this form of exercise may have been more efficient, perhaps because it enables patients to dispense with passive time listening to instructions or waiting for slower participants. The support of an exercise program leader has been identified as being valuable to mental health patients (55); however, a study of exercise preferences in substance abuse treatment revealed that men were equally as likely to favor exercising alone as with an instructor (56). In line with the first point in this discussion, this may imply that the diversity in exercise preferences should be kept in mind by clinic managers.

The finding that 57% of all patients met recommended levels of physical activity is encouraging, but unexpected. This indicates, during their stay at the hospital, the percentage of psychiatric patients with sufficient physical activity levels was comparable to that of the general Swiss population (57). In a recent meta-analysis, Schuch et al. (13) found that 68% of people with depression did not comply with the Centers for Disease Control and Prevention physical activity recommendation. Previous research also showed that initiating and maintaining regular exercise among psychiatric patients is challenging, because symptoms may interfere with patients' capacity to self-regulate health behaviors (58, 59). For instance, depressive symptoms may be associated with motivational and volitional deficits due to hopelessness, pessimism, loss of interest, and enjoyment in ordinary things, persistent low mood, low self-efficacy, limited capacity to plan due to impaired executive function, and a tendency to postpone tasks (58–60).

Accordingly, prior research suggested that compared to healthy controls, patients with major depression have limited exercise self-efficacy, stronger negative outcome expectations, reduced intentions to exercise, poor maintenance self-efficacy, and an increased perception of situational barriers (59, 61). Additionally, the presence of somatic co-morbidities may constitute a further barrier for people with psychiatric disorders to engage in sufficient physical activity (62). Given this background, the proportion of patients who met recommended physical activity levels was high in our sample, especially as we assessed physical activity objectively, which tends to yield lower scores compared to self-report (13, 14).

In our opinion, the high portion of sufficiently physically active patients in our study is due to the fact that all involved clinics are aware of the potential benefits of a physically active lifestyle, and two of these clinics were previously involved in a randomized controlled trial, in which the efficacy of exercise and sport therapy was tested. Thus, all clinics offered both structured and independent physical activity and exercise activities as part of their treatment modalities. Although these activities are not compulsory, it is strongly recommended to the patients to participate in these activities. In other words, it can be assumed that under these particular circumstances, in-patients might have been more physically active than can typically be expected for people with psychiatric disorders assessed in their natural living conditions.

In the present sample, only 45% (*n* = 48) of the patients reported that they do not engage in exercise and sport therapy. This portion is markedly below the 75% (roughly) estimated non-participation rate given by representatives of psychiatric clinics located in German-speaking Switzerland (39). By contrast, our findings also highlight that only 22% (*n* = 24) of all patients individually engage in exercise and sport activities; consequently, the majority of exercisers are making use of a program which will not be available to them following discharge. Given that the effects of structured exercise and sport therapy may dissipate if patients discontinue exercise participation after having been discharged for the hospital (63), a stronger focus should be placed on the sustainability of physical activity and exercise promotion (64). While preliminary evidence suggests that behavioral skills training through trained physical activity facilitators might constitute a viable way to improve physical activity levels among patients with psychiatric disorders (25, 65), this approach has yet to be tested in an in-patient setting.

The strengths of this study are that physical activity was assessed objectively and that previously established cut-offs were used to assess levels of moderate-to-vigorous physical activity levels (46). Furthermore, we performed a wear time validation to ensure that (estimated) weekly physical activity levels are based on at least five valid days with ≥90% wear-time. Moreover, face-to-face interviews were employed to ensure that all questions were correctly understood by the patients. We used an instrument specifically developed for psychiatric patients to gather information about the type of exercise and sport activities patients engaged in (49). Despite these strengths, the findings of the present study must be interpreted in light of several limitations: For instance, the focus of our study was on psychiatric in-patients who are living in a strongly externally structured environment. Future research should therefore investigate whether our findings also apply to out-patients. We further acknowledge that the sample size was relatively small in the present study, and that the majority of the patients was suffering from major depression. Larger and more diverse samples are needed to find out whether similar findings occur for patients suffering from different psychiatric disorders. Furthermore, no information was available how long the patients have been under treatment before their current stay at the hospital. Moreover, due to the limited sample size, we were not able to examine the relationships separately for each involved clinic. We also acknowledge that actigraphy is not able to accurately assess all physical activities such as bicycling or swimming (66). Finally, our study does not provide information about how exercise and sport participation during the hospital stay was associated with patients' physical activity levels after discharge, an issue that should be addressed in future research.

## Conclusion

The present study indicates that providing opportunities to engage in individual or structured exercise and sport activities increases the likelihood for psychiatric in-patients to accumulate sufficient levels of health-enhancing physical activity. Given that prolonged sedentary behavior has harmful effects on patients' physical and mental well-being, promoting exercise and sport activities is an important endeavor in psychiatric care. Our study shows that currently clinics succeed in involving between 50 and 60% of all patients in sufficient physical activity. While this is encouraging, more systematic efforts are needed to ensure that all patients get enough physical activity.

## Consent for publication

Consent for publication was obtained from the participants, collaborators, and co-authors.

## Availability of data and material

Can be requested for further analyses or transparency reasons from the corresponding author.

## Ethics statement

The study was approved by the local ethical committee (EKNZ, Approval number: 2016-01547) and all patients signed an informed consent to the study after receiving all relevant study information.

## Author contributions

JE, SE, MH, EH-T, CI, NS, SV, and MG developed the study design. CI, SE, SV, and NS coordinated the study at the three study sites. JE was responsible for the data assessment. MG, JE, and SE conducted the statistics. MG wrote the manuscript. All authors contributed to the data interpretation, and the internal revision of the manuscript draft. All authors approved the final draft version.

### Conflict of interest statement

The authors declare that the research was conducted in the absence of any commercial or financial relationships that could be construed as a potential conflict of interest.
